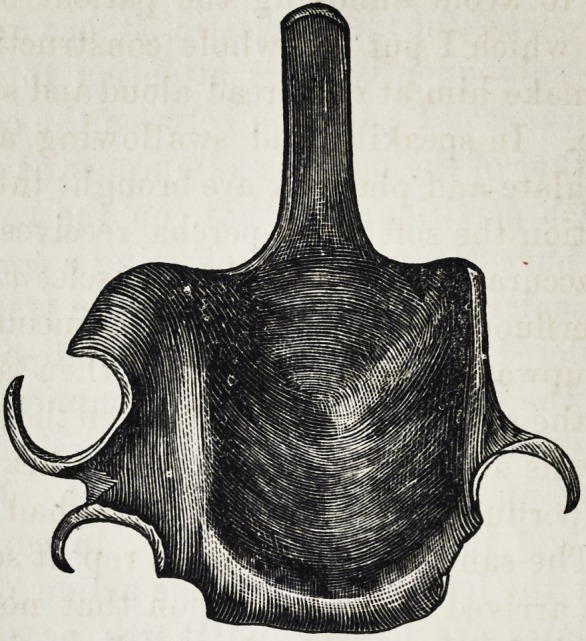# Artificial Palates

**Published:** 1868-05

**Authors:** Wilhelm Suersen

**Affiliations:** Berlin, Prussia.


### AMERICAN JOURNAL

OF

### DENTAL SCIENCE.

Vol. 11. THIRD SERIES—MAY, 1868. No. 1.

### ARTICLE I.

### *Artificial Palates.*

*A Lecture on the Restoration of a Distinct Utterance by Means of a New System of Artificial Palates to be Employed in all Cases of Congenital and Acquired Defects of the Palatine Organs.*

By Dr. WILHELM SUERSEN, Sen., Berlin, Prussia.

Delivered at Hamburg on the th of August, 1867, at the 8th Annual Meeting of the Central Association of German Dentists.*

GENTLEMEN :

Since the introduction of caoutchouc-teeth, nothing in our special branch has created more sensation than the Kingsley palates used in cases of inborn palate-slits. All of you are aware how much has, for several years past, been written on the subject in the whole cis- and transatlantic dentological literature and how fully the merits of Kingsley have on all sides been appreciated. To-day, I

*Having received from Dr. Suersen the whole of this lecture, extracts from which with engravings were published in the December number 1867 of Am. Jour. Dental Science, we present it to our readers (omitting the portions already published and referring to these at the proper places,) as a statement of the technical manipulations of this method of constructing artificial palates is necessary to make it perfectly intelligible. Eds.

have to submit to your notice, a new system of artificial palates, a system which I believe to be destined so assume the place of the Kingsley obturators. When I shall have laid my system theoretically and practically before you, it will be for you to pronounce your decision on it.

Before entering on the subject, I will only remark for those of my colleagues who have not made themselves specially acquainted with the Kingsley-palates, that the latter are composed of soft, moveable caoutchouc, supporting itself on the existing side-halves of the velum by which it is raised, whenever the levator veli palatini comes into action. My palates, in the first instance, are distinguished from the Kingsley-palates by the fact of their being made, not of soft, but of hard vulcanized caoutchouc. Now it is perfectly true that obturators for defects of the hard and of the soft palate have before this sometimes been made of the same material.

(The lecturer then mentions several cases in point but all of which essentially differ from his own construction. He then alludes to the subperiostical urano-plastic, introduced by Langenbeck in 1860,—an operation which, devised by Langenbeck with as much ingenuity as executed by him with consummate skill, in most cases affects the closing of the palate slits.) The lecturer then proceeds: But what is the success of this operation in regard to utterance? Unfortunately it is not such as might have been wished to so ingenious a method of operation. When I shall have explained to you the theory of the formation of sounds, on which my system is based, you will at once clearly see, why, even after the most successful operation, a satisfactory result for utterance is so frequently missed.*

These explanations, tending to show that the nasal sound is produced, if the partition between the cavity of

---

*For the propositions upon which this theory is based, the reader is referred to pages 374 to 376 of December No. 1867 of Amer. Journal of Dental Science.

the nose and that of the mouth cannot be effected, will easily account for the fact that even the most successful operation is frequently insufficient for effecting an essential improvement of utterance. The contraction of the long scar is in many cases so considerable, that the velum is too much shortened and therefore rendered unable to reach the back pharynx-wall.

The lecturer then proceeds to mention several methods of operation, which, have been suggested and executed by Passavant, for the purpose of improving the utterance, if, in spite of a successful urano-plastic, it has remained defective. He subsequently calls attention to the circumstance, that a *constant* partition between the cavity of the mouth, and that of the nose is as improper as the *constant absence* of such partition, but that, on the contrary, it is necessary to have the means of effecting a *transitory* partition by muscular action, and that such a transitory partition is rendered practicable by his palate-construction.

After this the lecturer narrates the manner in which he came by his construction. He had been desirous to submit the Kingsley-apparatus to a practical test. But the first patient, who got into his hands, had, in consequence of syphilis, lost the levator-veli, on the action of which the Kingsley-palates are based, and it was, accordingly, necessary to devise some other construction. The lecturer has brought this patient and two others with him from Berlin ; he produces eight different palates, all of which had been made for that first patient, and had subsequently been rejected, until he found the present simple construction which completely answers its purpose.*

The lecturer proceeds: I now request your attention for the technical process I employ in the construction of my palates. Above all things it is requisite to blunt the irritability of the soft palate, and of the pharynx, and to ob-

---

*For description of three cases see pages 376 and 377 December No. 1867 of Amer. Journal Dental Science.

viate the inclination for convulsive vomiting, where such inclination may exist. For this purpose excellent results will be attained by spreading a solution of alum over the pharynx and the velum, say about 1:32, to be applied several times a day. I then take a common wax-impression of the hard palate, in the same manner as for every set of artificial teeth ; after this I cause to be made a caoutchouc plate (eventually with artificial teeth, which, by means of cramps, is properly attached to teeth still existing, and in a backward direction, v. the following point) from the point at which the defect in the soft palate begins, terminates in a narrow and thin apophysis, freely projecting into the cavity of the mouth without in any way touching the soft parts. This apophysis, as we shall immediately see, has no other object than that of serving as a support to the real obturator for the soft palate. When the apparatus has so far proceeded, I cause it to be worn for a day or two, in order to convince myself of its giving no annoyance of any kind. If this is not the case, I proceed to the construction of the real obturator. The apophysis alluded to is perforated in some places, and provided with wire-knots for the purpose of affording a sufficient number of fixing points to the provisional gutta-percha mass to be attached to it. This precaution I consider necessary, for making quite sure lest the gutta should sooner or later come off, and by falling downwards, cause difficulty of breathing when the wearer of the apparatus feeds. I then form, by eyesight, around that apophysis provided with wire-knots, a mass of gutta-percha of about ¾ or 1 centimetre in height, corresponding as nearly as possible to the shape of the defect, and, at the same time, filling up the cavum pharyngo-palatinum. This mass I soften by heating it over a lamp fed with spirits of wine ; I dip it in water (merely to avoid annoying the patient by too much heat), after which I put the whole construction into his mouth and make him at once read aloud and swallow from time to time. In speaking and swallowing all the muscles of the palate and pharynx are brought into action and by their motion the soft gutta-percha receives a form exhibiting an accurate impression of the muscles *while in activity.* The superfluous gutta-percha is spontaneously squeezed away, both upwards and downwards. I then take the apparatus out of the patient's mouth ; I harden the gutta-percha mass by means of cold water, and with a heated knife remove the superfluous gutta-percha which had squeezed itself away. The same manipulations I repeat several times, until I have arrived at the conviction that no more gutta is pressed away by the continued muscular action, and that, consequently, the gutta-percha mass is no more too large. As a *test* of this I apply another simple proceeding to the back part of the mass lying in the cavum pharyngo-palatinum. By means of a small pair of pincers I produce little projections in the *vertical* back-levels of the softened gutta-percha mass ; I put the apparatus into the patient's mouth, but take it out again immediately, *without making the patient swallow or read.* If after this, the projections were squeezed flat, this would prove the gutta-percha mass to have touched the pharynx-wall, without the constrictor pharyngis having been in action. In this case the apparatus would, as a matter of course, be too large, and it would be necessary to take away more. As soon as it has thus been ascertained that the gutta-percha mass is not too large, I again make little projections ; I then put the construction again into the patient's mouth, and now immediately make him read aloud and swallow repeatedly. *By speaking and swallowing (that is, by the action of the constrictor pharyngis) those projections would be flattened, whereas, if the muscle alluded to had remained inactive, they would be left untouched.*

In this manner it may most reliably and in a way the most simple be ascertained, that the construction has assumed the correct dimensions.

The lower level—that which is turned towards the mouth—must, as I have already observed, lie so high as to reach the level of the velum platinum, when the latter has been lifted by the action of the levator veli. If, therefore, the side-halves of the velum hang, loosely down, the lower base of the back-part of the palate lies over it. If that basis lies too deep, it gives annoyance in swallowing and perhaps even excites an inclination to vomiting, if, on the other hand, it is placed too high, it will impede the closing of the palate gate, thus preventing the easy pronunciation of the palatic letters (k and g.)

The apparatus thus constructed I then make the patient wear for a few days, for the purpose of convincing myself that no annoyance is caused by it and, when I have finally arrived at that conviction, hard vulcanized caoutchouc is substituted for the provisional gutta-percha. The mode of proceeding : embedding a plaster-cast, removal of the gutta and filling up the vacant space which has arisen, with caoutchouc, I need not describe. It will be remembered that, in vulcanizing such thick pieces of caoutchouc, care must be taken that the temperature should not rise too rapidly, because in this case the caoutchouc would be apt to become porous and to swell out. It is more advisable to vulcanize with a lower temperature, but to continue the process for a longer time.

(On the following morning before the sitting was opened, the orator demonstrated practically all he had said, by constructing a palate for one of the three patients he had presented.)

Permit me now to say a few words on the difference between *acquired* and *inborn* defects of the palate, in reference to successful utterance. According to the experience I have hitherto made (I have, up to this moment, treated fifteen cases) I believe myself justified in affirming that all *acquired* defects in the last parts of the soft palate, however great or slight they may be, furnish a most satisfactory result. But how does the matter stand in reference to inborn slits of the palate? With them likewise the first condition sine qua non is, as a matter of course, the establishment of the physical relations, that is: the possibility of closing, by muscular action, the palate-valve between the cavity of the nose and that of the mouth. But this proceeding by no means suffices for such cases; for all the people laboring under such a defect, have never from their earliest youth been in the habit of pronouncing the single letters distinctly; on the contrary, their tongues have assumed the custom of making various incorrect supplementary movements. These incorrect movements it is necessary for those people to unlearn and they have to get used to the correct ones, before their utterance can acquire a normal condition. Such *unlearning* and *learning* is, of course, a task to be accomplished by the patients themselves. Of such like perverse habits, which I have had occasion to observe I will communicate a few instances: A female patient formed the letter k with hollowed tongue, coughing forth, as it were, a sound more similar to h than k. Until she had learned to close the palatic gate by arching the back of the tongue, the normal pronunciation of k was of course impossible to her. Two other patients were in the habit of arching the lingual root rather too much, in consequence of which the air, in passing through the back part of the pharynx, produced a rubbing noise which in the utterance of nearly every letter caused a guttural tone. Another patient, in pronouncing the letter s, (which, it must be remembered, is in a normal way produced by an *imperfect* closing of the lingual gate) laid the tip of his tongue *firmly* against the palate and front teeth, blowing the air through his nose and narrowing by the M. Compressor nasi his nostrils to such an extent that the air, in being pressed through them, made a hissing (s-like) noise. Again another patient, on inhaling the air, contracted the constrictor pharyngis so that the process of exhalation through the nose was impeded. In pronouncing the letter *m*, however, that muscle remained normally slack. The patient, after a short time left off that abuse, in consequence of my advice mentally to utter the letter *m* whenever he was exhaling. Another patient again had never blown his nose, because the mucus had at once run through the fissure into his mouth. When this was prevented by the obturator, he complained of a feeling of rheum, for the lower nasal passages were blocked up by the accumulating mucus ;—he had to learn to use a pocket-handkerchief.

These instances may suffice. They will justify the proposition: "*that in cases of inborn slits in the palate, the physical relations having been duly regulated, it depends on the intelligence and perseverance of the patient, how rapidly and to what extent his utterance may attain a normal condition.*"

To this is to be added another question, viz.: *At what age are we to apply the artificial palates?*

The reports on the Kingsley apparatus agree in this that favorable results have been observed only in children, but not in persons grown up. It is different with *my* system, as the patient presented, with an inborn defect of the palate, has proved to you. But I think myself justified in asserting, that with children the success appears more rapidly and becomes more complete. In children evil habits are not yet so inveterate, as they are in older persons; in youth we also learn more easily than we do at an age more advanced and it must therefore be set up as a general rule, *that the artificial palate should be applied at as early an age as possible;* that is, as soon as the child is intelligent enongh for wearing it and the existing teeth offer a sufficient number of fixing-points.

Another question: *how the palates are to be fixed if there are no teeth left.* I cannot answer from experience, because I never met with such a case till now. Nevertheless I believe I may remind you of the fact that the palates are placed above the halves of the velum palatinum and this position of itself affords them some support. Another point of support might perhaps be created by means of a projection leaning from the direction of the nasal cavity on the bony parts of the palate, in the same manner as Ramsey has done with the Kingsley-construction. If they should even then want firmness, this deficiency might be supplied by the application of spiral springs, such as are employed for all artificial sets of spring-teeth.

I will still emphatically observe that, as a matter of course, it will in the course of time be possible to devise and introduce many a little improvement of my palates. I shall myself find sufficient opportunity for further observation and I reserve it to myself to report on the subject at our next annual meeting. It now only remains to me to request all my esteemed colleagues, on their part not to keep from us any observations and experiences they may have made by that time.*

---

*For a comparison between this and the Kingsley palates, the reader is referred to page 377 of Decem. No. 1867 of Amer. Journal of Den. Science.

## Figures and Tables

**Figure f1:**